# Frailty and long-term survival of patients with ovarian cancer: A systematic review and meta-analysis

**DOI:** 10.3389/fonc.2022.1007834

**Published:** 2022-10-17

**Authors:** Kemin Li, Rutie Yin, Zhengyu Li

**Affiliations:** The Department of Obstetrics and Gynecology, West China Second University Hospital of Sichuan University, Chengdu, China

**Keywords:** frailty, ovarian cancer, survival, meta-analysis, geriatric assessment

## Abstract

**Background:**

Frailty has been related with poor prognosis of various diseases, including ovarian cancer. We performed a systematic review and meta-analysis to evaluate the association between frailty and long-term survival of patients with ovarian cancer.

**Methods:**

Relevant cohort studies were retrieved by search of PubMed, Embase, Cochrane’s Library, and Web of Science electronic databases. Two authors independently performed literature search, data collection, and statistical analyses. A random-effect model incorporating the possible influence of heterogeneity was used to pool the results.

**Results:**

Nine cohort studies including 2497 women with confirmed diagnosis of ovarian cancer contributed to the meta-analysis, and 536 (21.5%) of them were with high frailty. The median follow-up durations varied between 24 and 69 months. Compared to patients with low or non-frailty, OC patients with high frailty were associated with poor overall survival (risk ratio [RR]: 1.61, 95% confidence interval [CI]: 1.41 to 1.85, p < 0.001; I^2^ = 0%) and progression-free survival (RR: 1.51, 95% CI: 1.20 to 1.89, p < 0.001; I^2^ = 0%). Subgroup analyses according to study design, cancer stage, age of patients, scales for frailty evaluation, follow-up duration, and quality score of the included study showed consistent association between high frailty and poor overall survival in women with ovarian cancer (p for subgroup effects all < 0.05). After considering GRADE criteria for strength of the evidence, it was rated low for both the two outcomes.

**Conclusion:**

High frailty may be an independent risk factor of poor survival in women with ovarian cancer. Evaluating frailty may be important for predicting the prognosis and determining the optimal anticancer treatments in women with ovarian cancer.

**Systematic Review Registration:**

https://inplasy.com/, identifier INPLASY202290028.

## Introduction

Currently, ovarian cancer (OC) ranks the fifth leading cause of cancer-related mortality of the global population (1, 2). The incidence of OC is generally lower compared to other gynecological cancers (3). However, because the symptoms of OC tend to be non-specific and the effective screening methods for OC are still lacked, patients with OC are likely to be diagnosed at advanced stage, which may be an important reason for the poor prognosis of these patients (4, 5). Surgical resection is the preferred treatment for OC if diagnosed early (6). Although emerging therapies such as poly (ADP-ribose) polymerase (PARP) inhibitors are being developed in maintenance and recurrence treatment settings of epithelial OC with BRCA 1-2 gene mutation (7), for most patients with advanced OC, tumor debulking followed by adjunctive therapy is recommended (6, 8). Many factors have been proposed to influence the prognosis of patients with OC, such as age, cancer stage, grade, histological type, and anticancer treatments etc. (8, 9). However, for some patients with OC, prognostic prediction remains difficult (10, 11), which highlights the importance of the identification of new prognostic factors in patients with OC.

Recent advances in oncogeriatrics suggested the important role of geriatric evaluation for frailty in the risk stratification and optimized management of patients with cancer (12). By definition, frailty refers to a state of age-related decline in biological reserve, decreased ability to maintain physiological balance and increased vulnerability to adverse health events (13, 14). It has been proposed that frailty may be a key factor which affects the therapeutic efficacy and toxicity of anticancer modalities (15, 16). Accordingly, high frailty has been shown to be a predictor of poor survival in patients with some types of cancers, such as lung cancer (17), prostate cancer (18), colorectal cancer (19), and other digestive system tumors (20). However, studies evaluating the correlation between frailty and survival of patients with OC showed inconsistent results (21–29). Some studies suggested that high frailty was associated with poor survival of these patients (22, 24, 25, 27–29), while others failed to show a significant association (21, 23, 26). Therefore, we performed a systematical review and meta-analysis to comprehensively investigate the relationship between frailty and survival of patients with OC.

## Materials and methods

The Preferred Reporting Items for Systematic Reviews and Meta-Analyses (PRISMA) statement (30, 31) was followed in designing, performing, and reporting the meta-analysis was in accordance with the recommendations of the Cochrane’s Handbook (32) guideline. The protocol of the meta-analysis has been registered at INPLASY (International Platform of Registered Systematic Review and Meta-analysis Protocols, https://inplasy.com/) with the registration number of INPLASY202290028).

### Literature retrieving

Studies were retrieved by search of PubMed, Embase, Cochrane’s Library and Web of Science electronic databases from the inception to April 2, 2022. A combined search term was used, including (1) “frailty” OR “frail”; (2) “ovarian” OR “ovary”; and (3) “cancer” OR “carcinoma” OR “malignancy” OR “tumor” OR “neoplasm”. The search was limited to human studies published in full-length articles. No restriction was applied regarding the language of publication. As a supplementation, we manually checked the citations of the relevant original and review articles for possible studies of interest.

### Study selection

The PICOS criteria were used for study inclusion.

**P (patients)**: Adult patients with histologically confirmed diagnosis of OC, regardless of the cancer stage or treatments.

**I (exposure)**: Patients with high frailty at admission. Methods and criteria for defining patients with frailty were consistent with the modalities used in the original studies.

**C (control)**: Patients with low or non-frailty at admission. The evaluating tools and criteria for the frailty were consistent with those applied among the included studies.

**O (outcomes)**: the primary outcome was overall survival (OS), and the secondary outcomes were progression-free survival (PFS), compared between OC patients with high versus low or non-frailty. Generally, OS was defined as the time elapsed from treatment and to the date of death from any cause, while PFS was defined as the interval between initiation of the treatment and the first recurrence or progression event.

**S (study design)**: cohort studies, including prospective and retrospective cohorts;

Reviews, preclinical studies, studies including non-OC patients, studies that did not evaluate frailty, or studies that did not report the survival outcomes were excluded. In addition, studies with follow-up duration within months were also excluded because we did not aim to evaluate the immediate influence of frailty on mortality of patients with OC.

### Data collection and quality assessing

Two independent authors conducted literature search and analysis, data collection, and study quality assessment separately. If discrepancies were encountered, they were resolved by discussion with the third author to reach consensus. Data of study information, design characteristics (prospective or retrospective), patient demographic factors, cancer stage, main treatments, scales for the evaluation of frailty, follow-up durations, outcomes reported, and variables adjusted in the regression model for the analysis of the association between frailty and survival outcomes were collected. Quality of the included studies was evaluated *via* the Newcastle–Ottawa Scale (33) with scoring regarding the criteria for participant selection, comparability of the groups, and the validity of the outcomes. The scale ranged between 1-9 stars, with larger number of stars presenting higher study quality.

### Statistical analyses

The main objective was to determine the relative risks of OS and PFS of OC patients with high versus low or non-frailty, which were presented as risk ratios (RRs) and the confidence intervals (CIs). Using the 95% CIs or p values, data of RRs and the standard errors (SEs) could be calculated, and a subsequent logarithmical transformation was conducted to keep stabilized variance and normalized distribution. Between study heterogeneity was estimated with the Cochrane’s Q test and the I^2^ statistic (34). The between-study heterogeneity was classed as mild (I^2^ < 25%), moderate (I^2^ 25%~75%), and high (I^2^ >75%) according to the Cochrane’s Handbook (32). A random-effect model with the DerSimonian & Laird approach was applied to pool the results after incorporating of possible between-study heterogeneity (32). Influencing analyses by excluding one cohort at a time were performed to evaluate the stability of the results (35). Subgroup analyses were also performed to explore the influences of study characteristics on the outcome. By construction of the funnel plots, the publication bias was estimated based on the visual judgement of the symmetry of the plots, supplemented with the Egger’s regression asymmetry test (36). The Grading of recommendation, assessment, development and evaluation (GRADE) methodology was used to evaluate the quality of the body of retrieved evidence (GRADEpro, https://gdt.gradepro.org/app/#projects). The RevMan (Version 5.1; Cochrane Collaboration, Oxford, UK) and Stata (Version 17.0; Stata Corporation, College Station, TX) software were applied for these analyses, and a p < 0.05 suggests statistical significance.

## Results

### Studies obtained

**Figure 1:**
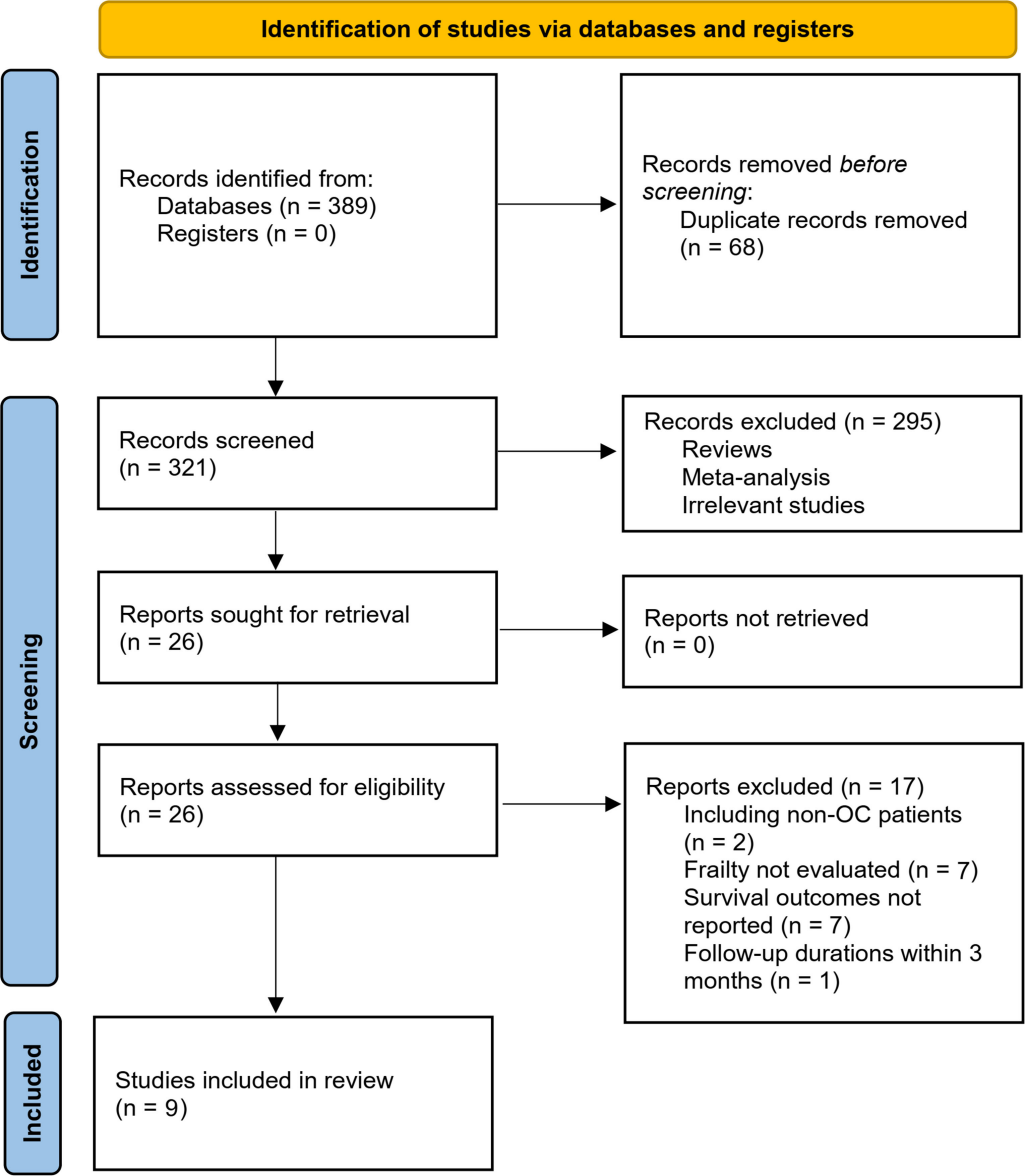
Summarized process of literature search and study identification.

**Figure 1** shows the process of literature analysis. In short, the initial search of the databases retrieved 389 articles, and 321 were left after excluding the duplicated records. Then, an additional 295 articles were excluded since the contents of the titles and abstracts indicated that they were not relevant to the aim of the meta-analysis, which made a total of 26 studies for the full-text review. Finally, after excluding 17 studies through full-text review, nine studies (21–29) were included. The reasons for the removing of the 17 studies are also presented in **Figure 1**.

### Characteristics of the included studies

As presented in **Table 1**, nine cohort studies, including two prospective (23, 28) and seven retrospective cohorts (21, 22, 24–27, 29), with 2497 patients with OC contributed to the meta-analysis. These studies were published between 2017 and 2022, and performed in the United States (22, 23, 25, 27, 29), Italy (21), France (24), and Germany (26, 28). All of the studies included patients with confirmed diagnosis of OC, and four studies included older patients only (21, 24, 26, 27). Five studies included patients with stage I-IV OC (21, 23, 24, 26, 28), three studies included patients with stage III-IV OC (22, 25, 27), while the remaining one study included patients with stage II-IV OC (29). Most of the included patients received surgical treatment for OC. Various scales were used for the evaluation of frailty, such as the frailty deficit index (22, 25, 28), the modified frailty index (mFI) (21, 24, 29), the Fried frailty phenotype (23), G-8 score (26), and the memorial Sloan Kettering Frailty Index (27). Accordingly, 536 (21.5%) of the included patients were with high frailty at admission. Among the included studies, three of them compared the survival between patients with high versus low frailty (21, 24, 29), and the other six compared the survival between patients with high versus non-frailty (22, 23, 25–28). The median follow-up durations varied from 24 to 69 months. The OS was reported in all of the nine studies (21–29), while the outcome of PFS was reported in four studies (24–26, 29). Univariate analyses were performed in two studies (21, 24) when the association between frailty and survival outcome was analyzed, while multivariate analyses were performed in seven studies (22, 23, 25–29). Variables such as age, cancer stage, grade, histological type, and concurrent treatments were adjusted in the multivariate models. The NOS of the included studies were 6 to 9 stars, suggesting moderate to good study quality (**Table 2**).

**Table 1:** Characteristics of the included studies.

Study	Location	Design	Diagnosis	FIGO stage	Main treatment	Number of patients	Age (years)	Tools for the diagnosis of frailty	Number of women with high frailty	Median follow-up duration (months)	Outcomes	Variables adjusted
Kumar 2017	USA	RC	Patients with OC receiving primary debulking surgery	III-IV	Surgery	535	Mean: 64.3	Frailty deficit index	131	40	OS	Age, preoperative albumin, grade, stage, histology, and residual disease
Ferrero 2017	Italy	RC	Older patients with OC	I-IV	Surgery or chemotherapy	78	70~89, median: 75.5	Modified frailty index	23	60	OS	None
Narasimhulu 2020	USA	RC	Patients with OC receiving primary debulking surgery and adjuvant chemotherapy	III-IV	Surgery and chemotherapy	169	Mean: 63.3	Frailty deficit index	29	30	OS and PFS	Age, stage, serous histology, and residual disease
Cespedes 2020	USA	PC	Patients with OC	I-IV	NR	286	50~79	Fried frailty phenotype	37	69	OS	Age, stage, ethnicity, BMI, smoking, educational attainment, comorbidities, and any family history of cancer
Dion 2020	France	RC	Older patients with OC	I-IV	Surgery or chemotherapy	147	70~99, median: 81	Modified frailty index	65	60	OS and PFS	None
Anic 2021	Germany	RC	Older patients with OC receiving debulking surgery	I-IV	Surgery	116	60~, mean: 70.9	G-8 score	54	30	OS and PFS	Age, stage, histologic type, grade, PS, comorbidities, residual disease, and concurrent treatments
Guelhan 2021	Germany	PC	Patients underwent surgeries for OC	I-IV	Surgery	144	18~87, median: 58	Frailty deficit index	47	38	OS	Age, stage, PS, albumin, surgical complexity, and residual disease
Filippova 2021	USA	RC	Older patients with OC receiving surgical treatment	III-IV	Surgery	430	72~79, median: 75	The memorial Sloan Kettering Frailty Index	74	24	OS	Age, stage, grade, postoperative complications, and concurrent treatments
Handley 2022	USA	RC	Patients with OC	II-IV	Surgery or chemotherapy	592	22~89, median: 64	Modified frailty index	76	32	OS and PFS	Age, stage, BRCA status, and residual disease

NR, not reported; OC, ovarian cancer; FIGO, the International Federation of Gynecology and Obstetrics; RC, retrospective cohort; PC, prospective cohort; OS, overall survival; PFS, progression-free survival; BMI, body mass index; PS, performance status; BRCA, breast cancer susceptibility gene.

**Table 2:** Quality evaluation of the included studies.

Study	Representativeness of the exposed cohort	Selection of the non-exposed cohort	Ascertainment of exposure	Outcome not present at baseline	Control for age	Control for other confounding factors	Assessment of outcome	Enough long follow-up duration	Adequacy of follow-up of cohorts	Total
Kumar 2017	0	1	1	1	1	1	1	1	1	8
Ferrero 2017	0	1	1	1	0	0	1	1	1	6
Narasimhulu 2020	0	1	1	1	1	1	1	1	1	8
Cespedes 2020	1	1	1	1	1	1	1	1	1	9
Dion 2020	0	1	1	1	0	0	1	1	1	6
Anic 2021	0	1	1	1	1	1	1	1	1	8
Guelhan 2021	1	1	1	1	1	1	1	1	1	9
Filippova 2021	0	1	1	1	1	1	1	1	1	8
Handley 2022	0	1	1	1	1	1	1	1	1	8

### Frailty and survival of patients with OC

**Figure 2:**
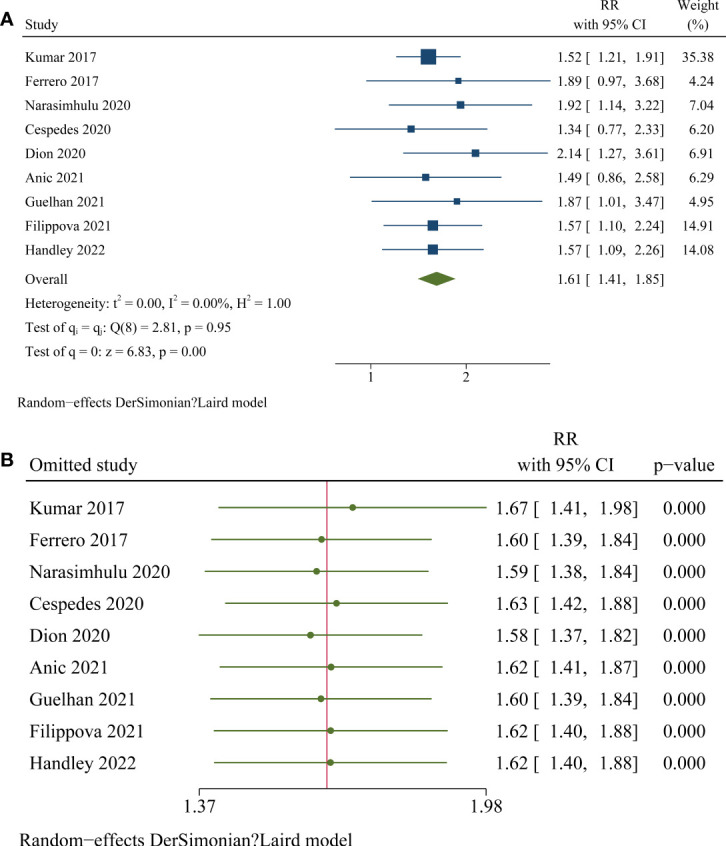
Forest plots for the meta-analyses regarding the association between high frailty and OS in patients with OC. **(A)**, overall meta-analysis; **(B)**, influencing analysis by omitting one study at a time.

**Figure 3:**
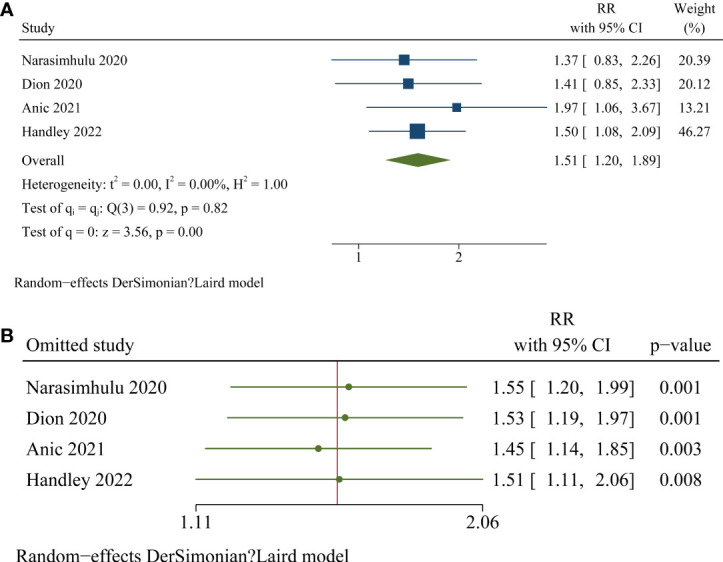
Forest plots for the meta-analyses regarding the association between high frailty and PFS in patients with OC. **(A)**, overall meta-analysis; **(B)**, influencing analysis by omitting one study at a time.

Pooled results with nine studies (21–29) showed that OC patients with high frailty were associated with poor OS compared to low or non-frail patients (RR: 1.61, 95% CI: 1.41 to 1.85, p < 0.001; **Figure 2A**) with no significant heterogeneity (p for Cochrane’s Q test = 0.95, I^2^ = 0%). Influencing analyses by omitting one study at a time showed consistent results (RR: 1.58 to 1.67, p all < 0.001; **Figure 2B**). Subgroup analyses according to study design, cancer stage, age, scale for frailty evaluation, follow-up duration, and quality score of the included study showed consistent association between frailty and poor overall survival in women with ovarian cancer (**Table 3**, p for subgroup effects all < 0.05). Specifically, subgroup analyses showed consistent association between studies comparing patients with high frailty versus patients with low frailty (RR: 1.76, 95% CI: 1.34 to 2.32, p < 0.001; I^2^ = 0%) and studies compared to patients with non-frailty (RR: 1.57, 95% CI: 1.34 to 1.84, p < 0.001; I^2^ = 0%; **Table 3**). In addition, pooled results with four studies (24–26, 29) also showed that high frailty was associated with poor PFS in patients with OC (RR: 1.51, 95% CI: 1.20 to 1.89, p < 0.001; **Figure 3A**) with no significant heterogeneity (Cochrane’s Q test = 0.82, I^2^ = 0%). Similarly, influencing analyses by omitting one study at a time showed consistent results (RR: 1.45 to 1.55, p all < 0.01; **Figure 3B**).

**Table 3:** Subgroup analyses for the association between frailty and overall survival in patients with OC.

Study characteristics	Datasets number	RR (95% CI)	I^2^	P for subgroup effect	P for subgroup difference
Design					
Prospective	2	1.55 [1.03, 2.34]	0%	0.04	
Retrospective	7	1.62 [1.40, 1.88]	0%	< 0.001	0.85
FIGO stage					
I-IV	5	1.71 [1.32, 2.21]	0%	< 0.001	
II-IV	1	1.57 [1.09, 2.26]	NA	0.02	
III-IV	3	1.58 [1.32, 1.89]	0%	< 0.001	0.87
Age					
60 years or above	4	1.70 [1.34, 2.17]	0%	< 0.001	
All adult patients	5	1.57 [1.33, 1.86]	0%	< 0.001	0.60
Frailty evaluation					
Frailty deficit index	3	1.61 [1.32, 1.96]	0%	< 0.001	
Modified frailty index	3	1.76 [1.34, 2.32]	0%	< 0.001	
Others	3	1.50 [1.15, 1.95]	0%	0.003	0.70
Selection of controls					
High versus low frailty	3	1.76 [1.34, 2.32]	0%	< 0.001	
High versus non-frailty	6	1.57 [1.34, 1.84]	0%	< 0.001	0.46
Follow-up duration					
< 40 months	5	1.64 [1.34, 2.00]	0%	< 0.001	
40 months or longer	4	1.59 [1.32, 1.93]	0%	< 0.001	0.85
Analytic model					
Univariate	2	2.04 [1.35, 3.08]	0%	< 0.001	
Multivariate	7	1.57 [1.35, 1.81]	0%	< 0.001	0.23
Quality score					
6	2	2.04 [1.35, 3.08]	0%	< 0.001	
8	5	1.57 [1.34, 1.83]	0%	< 0.001	
9	2	1.55 [1.03, 2.34]	0%	0.04	0.49

RR, risk ratio; CI, confidence interval; NA, not applicable; OC, ovarian cancer; FIGO, the International Federation of Gynecology and Obstetrics.

### Publication bias

**Figure 4:**
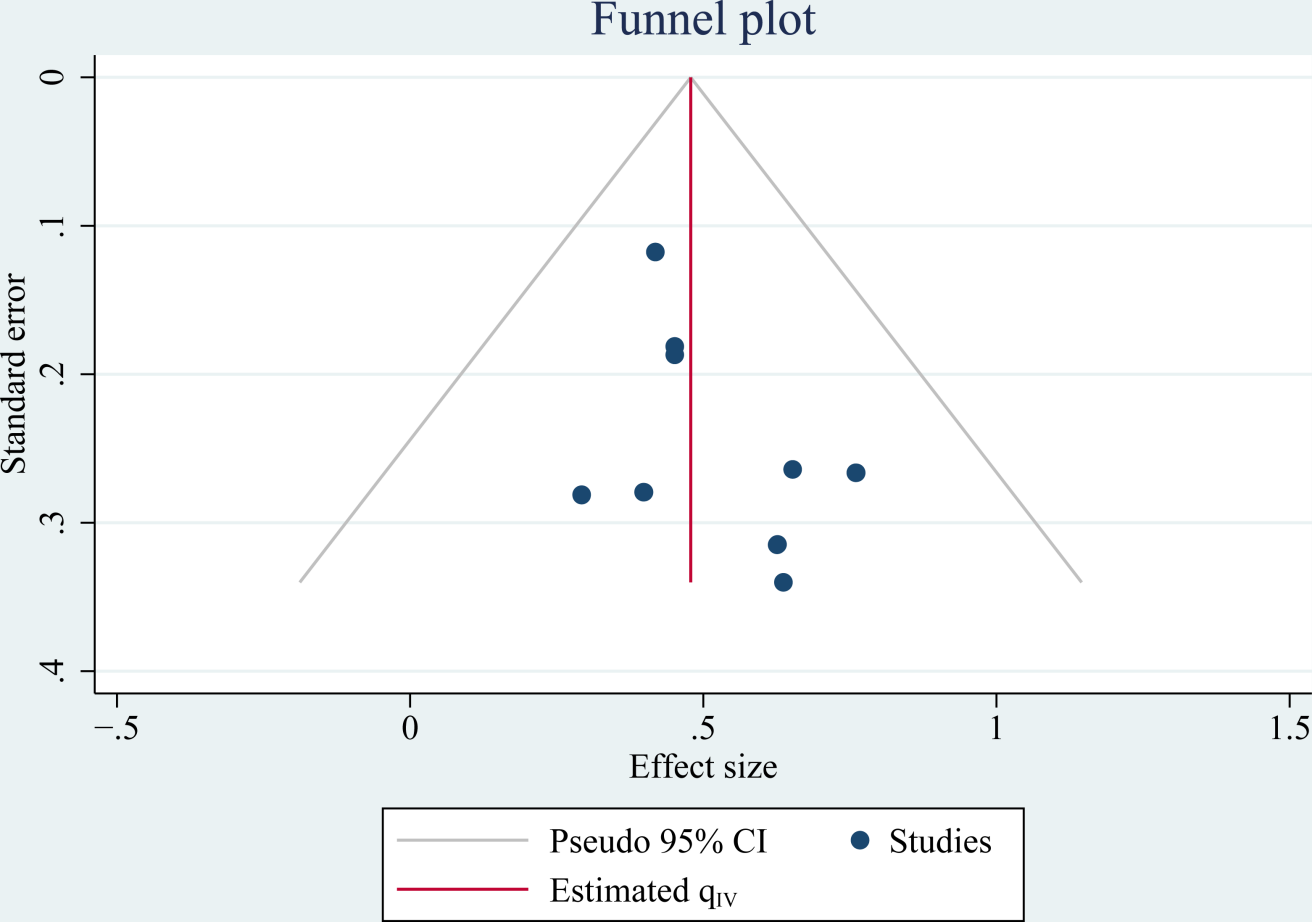
Funnel plots for the publication bias underlying the meta-analysis regarding the association between high frailty and OS in patients with OC.

**Figure 4** display the funnel plots for the meta-analysis of the association between frailty and OS in patients with OC. Visual inspection revealed symmetry of the plots, reflecting low risks of publication biases. The Egger’s regression tests also showed low risk of publication bias (p = 0.48). The publication bias for the meta-analysis of the association between frailty and PFS was difficult to determine because only four studies were included.

### Evaluation of the evidence

**Table 4** provides an overview of the GRADE assessment for the association between frailty and survival of patients with OC. The level of evidence was rated generally low for both the outcomes of OS and PFS.

**Table 4:** Certainty of evidence by GRADE criteria.

	Frailty and OS in OC	Frailty and PFS in OC
No. of studies	9	4
Downgrade quality of evidence		
Risk of bias	No	No
Inconsistency	No	No
Indirectness	No	No
Imprecision	No	No
Publication bias	No	No
Upgrade quality of evidence		
Large effect	No	No
Plausible confounding	No ^a^	No ^a^
Dose–response	No	No
Overall quality of evidence	Low	Low

a, Despite better quality studies provided less heterogeneity across results.

GRADE, Grading of recommendation, assessment, development and evaluation; OS, overall survival; PFS, progression-free survival; OC, ovarian cancer.

## Discussion

This systematical review and meta-analysis integrated the findings of nine cohort studies, and the pooled results showed that OC patients with high frailty were associated with poor long-term survival. The results were consistent for outcomes of OS and FPS. Moreover, subsequent sensitivity analyses by omitting one study at a time showed consistent results. In addition, for the meta-analysis of the outcome of OS, subgroup analyses showed that the association between frailty and poor OS in patients with OC was robust and not significantly affected by study characteristics such as study design, cancer stage, patient age, frailty evaluation scale, level of frailty of the controls, follow-up durations, analytic models, and study quality scores. Taken together, these results indicated that frailty may be an important risk factor of the poor long-term survival in patients with OC.

To the best of our knowledge, this is the first systematical review and meta-analysis which evaluated the association between frailty and long-term survival outcomes in patients with OC. The strengths of the meta-analysis included extensive literature searching to incorporate the up-to-date literature evidence, including cohort studies to indicate a longitudinal relationship between frailty and poor survival of patients with OC, and performing multiple sensitivity and subgroup analyses to confirm the robustness of the findings. The findings of the meta-analysis indicated a potential association between high frailty and poor survival of patients with OC. The mechanisms underlying the association may be multifactorial. Biologically, frailty has been associated with chronic inflammation and immunosenescence (37), which has also been involved in the carcinogenesis and invasion of the cancers (38). Besides, people with frailty were at higher risk for sarcopenia (39) and cancer-related cachexia (40), both of which have been related to a poor long-term survival of patients with OC (41). In addition, previous studies have shown that compared to non-frailty patients, OC patients with frailty were more likely to have postoperative morbidity (42, 43) and mortality (44), ICU admission (45), and intolerance for the standard-of-care chemotherapy (46) compared to the none-frailty patients, all of which may lead to poor long-term survival in these patients. Subgroup analyses according to multiple study or patient characteristics showed a consistent association between frailty and poor OS in patients with OC, suggesting a universal role of frailty as a predictor of poor prognosis in OC patients with different ages and cancer stages. Specifically, multiple evaluating scales were used for the evaluation of frailty among the included studies, and the results of subgroup analysis showed a consistent association in different scales. A previous large-scale study in community-dwelling elderly population with different frailty tools showed a similar capacity to detect frailty and a similar prognostic impact (47). Furthermore, the prognostic impact of frailty was also suggested to be similar among the oncologic patients (48). However, further large-scale studies are needed to determine if the prognostic efficacies of different frailty tools are similar in patients with OC.

Results of the meta-analysis also highlighted the significance of geriatric evaluation for frailty in patients with cancer, including those with OC. Besides the importance in prognostic prediction, identification of frailty patients with cancer may be important for the determination of appropriate anticancer modalities in these patients, considering these patients are less tolerable for surgeries and adjuvant therapies and more likely to develop complications and toxicity events. Moreover, geriatric co-management in cancer patients with frailty is also clinical significance, which may increase their tolerability to anticancer treatments and finally improve the clinical outcomes. For example, a previous study showed that in older women with advanced OC and frailty, preoperative/postoperative geriatric and surgical co-management may improve their tolerance to cytoreductive surgery and subsequent postoperative outcomes (49). Similarly, a recent clinical study confirmed that a geriatric assessment and intervention could reduce the serious toxic effects from cancer treatment in older patients with advanced cancer (50). Finally, besides identification of frailty patients with cancer, geriatric evaluation in patients with cancer is also useful to identify the “fit” older cancer patients, who could receive standard anticancer treatment similar to the young patients (51, 52). Collectively, results of the meta-analysis support the incorporating of geriatric evaluation for frailty into the routine management of patients with cancer.

Our study also has limitations. Firstly, as mentioned above, different tools for frailty assessment were used among the included studies, which may contribute to the clinical heterogeneity of the meta-analysis. Besides, the definition of frailty was different even among studies using the same methods. For example, for the three studies using mFI as the evaluating tool for frailty, a high frailty was defined as mFI ≥ 4 (21), a modified Charlson Comorbidity Index [mCCI] score >3 (24), and the adjusted modified frailty index score (amFI) ≥ 2 (29), respectively. Although no statistical heterogeneity was observed and subgroup analyses according to the frailty tools showed consistent results, studies are needed to determine the optimal scales and cutoff values for the determination of cancer patients with high frailty versus low or non-frailty. Moreover, we could not determine if the different histological type of OC may affect the association between frailty and survival because none of the included studies reported the stratified data according to the histological type of the cancer. Studies are warranted in the future to address this issue. Finally, as a meta-analysis based on observational studies, we could not confirm a causative relationship between frailty and poor survival of patients with OC. Clinical studies are needed to determine if intervention targeting frailty in patients with OC could improve the long-term clinical outcomes of these patients.

To sum up, results of this meta-analysis indicate that high frailty may be a risk factor of poor survival in patients with OC. These results highlight the importance of geriatric evaluation for frailty in patients with OC, which may be important for prognostic prediction and determination of the appropriate anticancer treatments for these patients.

## Data availability statement

The original contributions presented in the study are included in the article/Supplementary Material. Further inquiries can be directed to the corresponding author.

## Author contributions

KL and ZL designed the study. KL and RY performed database search, literature review, study quality evaluation, and data collection. KL and ZL performed statistics and interpreted the results. KL drafted the manuscript. ZL critically revised the manuscript. All authors approved the submission of the manuscript.

## Conflict of interest

The authors declare that the research was conducted in the absence of any commercial or financial relationships that could be construed as a potential conflict of interest.

## Publisher’s note

All claims expressed in this article are solely those of the authors and do not necessarily represent those of their affiliated organizations, or those of the publisher, the editors and the reviewers. Any product that may be evaluated in this article, or claim that may be made by its manufacturer, is not guaranteed or endorsed by the publisher.
